# Supplementary material for: Dendritic Polyglycerol Sulfate Reduces Inflammation Through Inhibition of the HMGB1/RAGE Axis in RAW 264.7 Macrophages
Source: Int J Mol Sci. 2025 Oct 27;26(21):10440. doi: 10.3390/ijms262110440 (PMC12607995; doi:10.3390/ijms262110440)
Supplement: Supplementary file 1 [file ijms-26-10440-s001.zip › ijms-3882784-supplementary.pdf]

Supplementary Information: Dendritic Polyglycerol sulfate reduces inflammation through inhibition of the HMGB1/RAGE axis in RAW 264.7 macrophages.

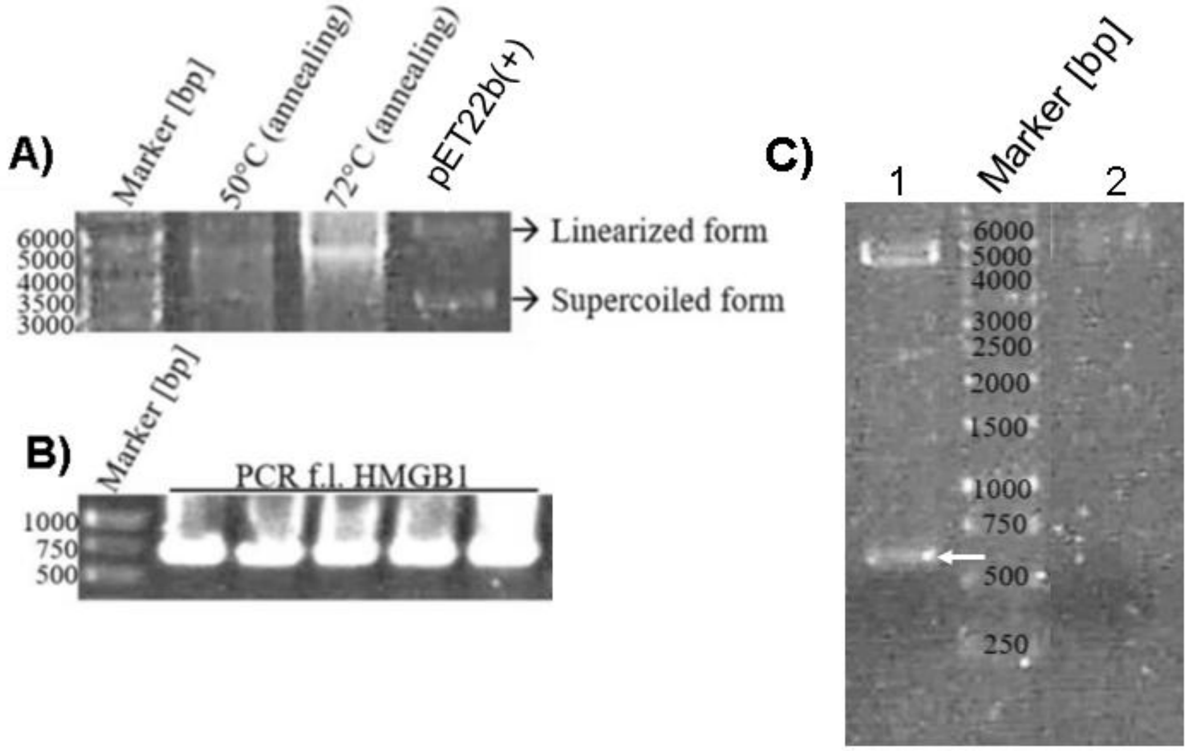

Figure S1. Modifying the pET22b(+) vector and generating HMGB1-specific PCR products with restriction analysis of selected clones. A) PCR products of the insertion of Serine-Glycine-Serine-Histidine-Histidine in the pET22b(+)vector analyzed on a 1% agarose gel. Two different annealing temperatures, 50 °C and 72 °C, were tested, whereas 72 °C yields a more representative band. As a vector control, the pet22b(+) vector was loaded at 1 µg, showing the linearized and supercoiled species. B) PCR products of HMGB1 (645 bp) were separated on a 1.5% agarose gel. C) Restriction digestion analyses of HMGB1 in mod. pET22b(+) analyzed on a 1.5% agarose gel 1 *NdeI/XhoI digestion*, 2 undigested.

A)

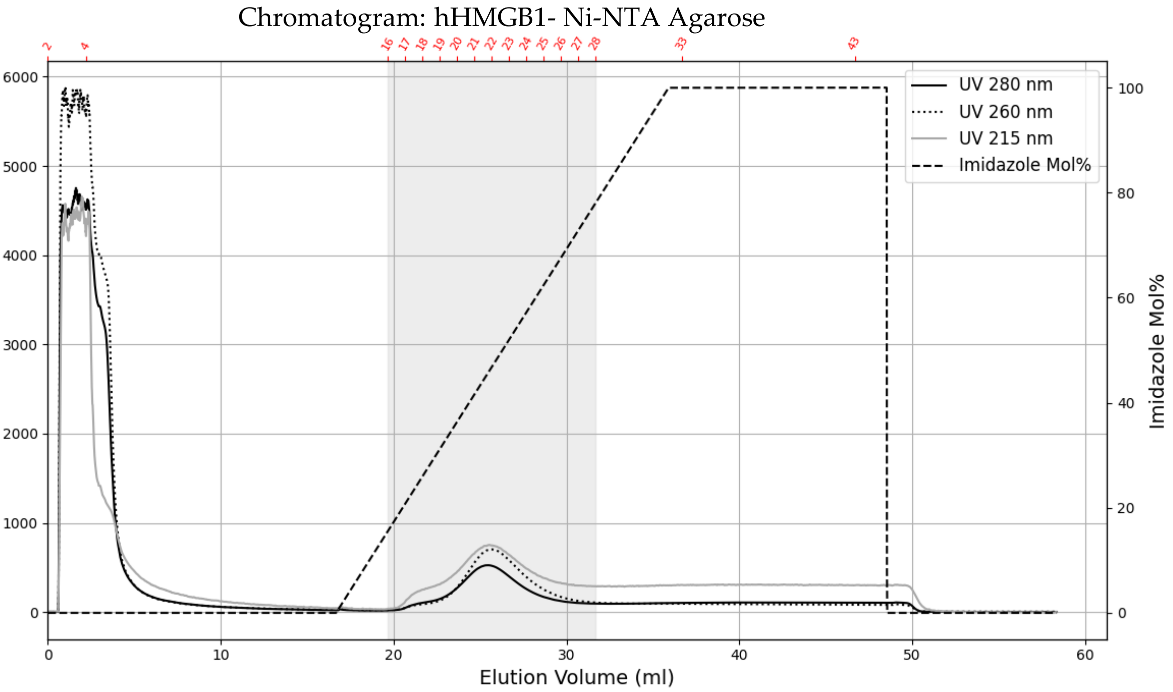
Chromatogram: hHMGB1- Ni-NTA Agarose
UV 280 nm
UV 260 nm
UV 215 nm
Imidazole Mol%
Absorbance (mAU)
Imidazole Mol%
Elution Volume (ml)

B)

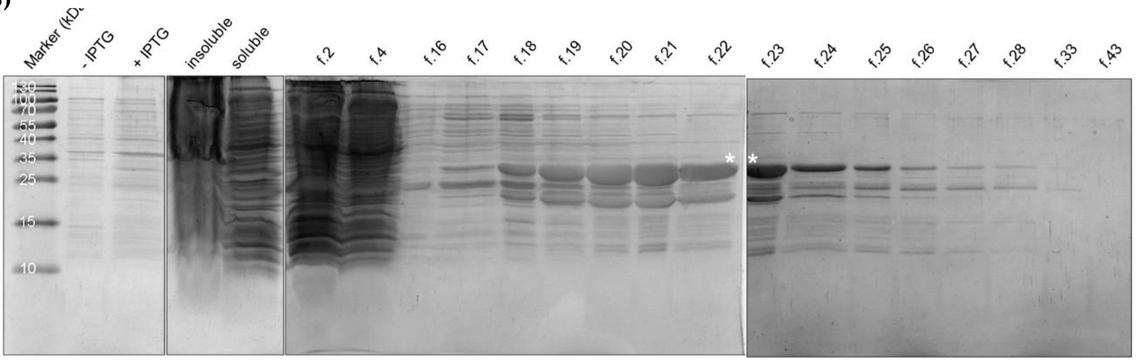
- IPTG
+ IPTG
insoluble
soluble
f.2
f.4
f.16
f.17
f.18
f.19
f.20
f.21
f.22
f.23
f.24
f.25
f.26
f.27
f.28
f.33
f.43

C)

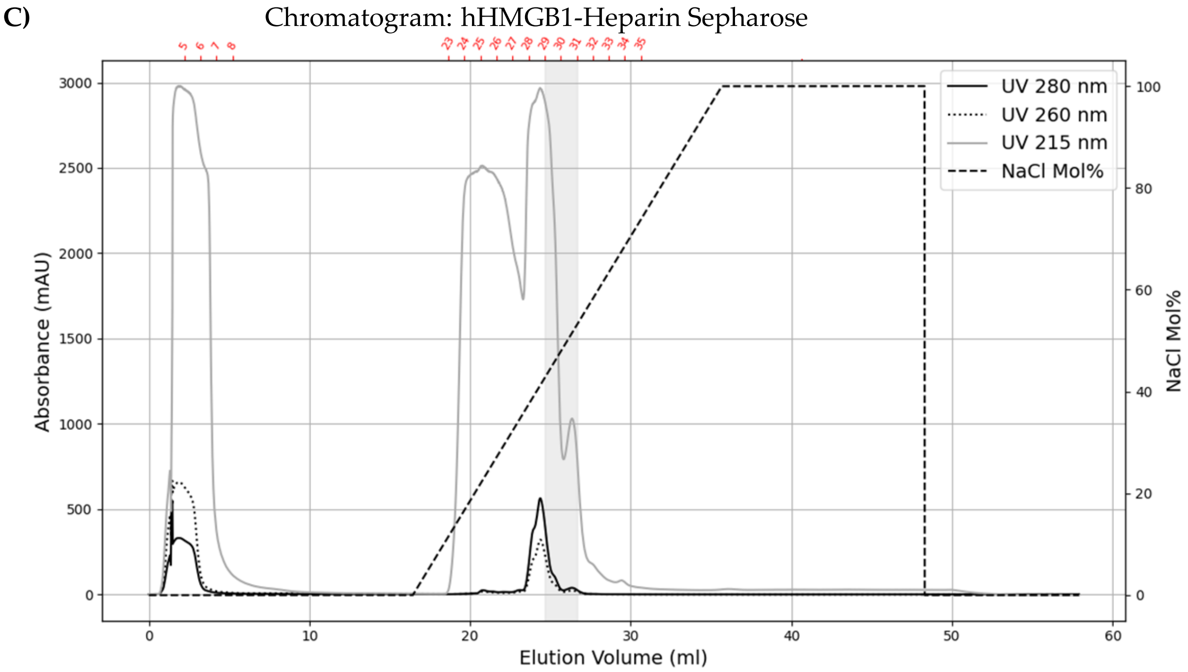
Chromatogram: hHMGB1-Heparin Sepharose
UV 280 nm
UV 260 nm
UV 215 nm
NaCl Mol%
Absorbance (mAU)
NaCl Mol%
Elution Volume (ml)

D)

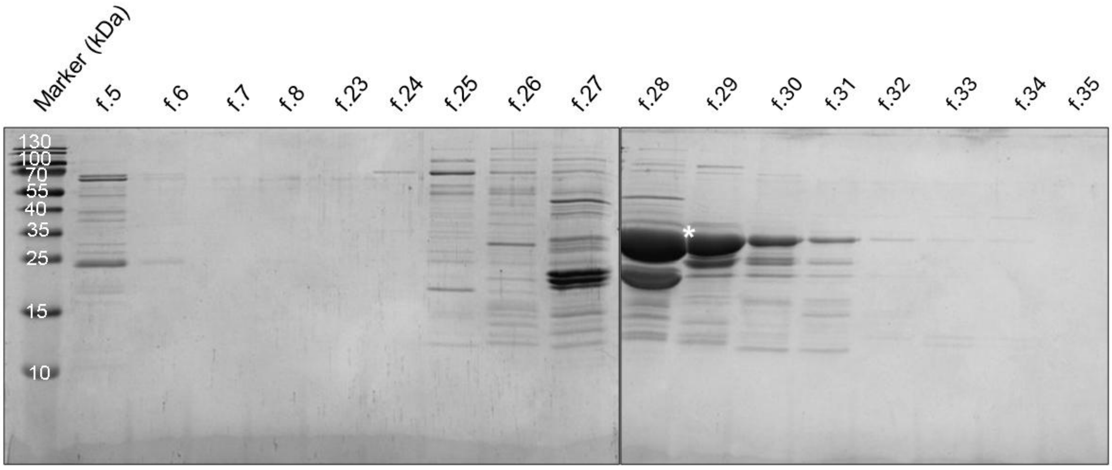

Figure S2. Recombinant expression and purification of HMGB1. A) Chromatogram of soluble extract on Ni-NTA agarose (100 Mol% imidazole elution buffer equals a 500 nM concentration). The chromatography was followed by absorbance measurements, and the collected fractions are indicated by red numbers. Fractions 16-28 were pooled and further processed. B) SDS-PAGE of cell lysates (-/+ IPTG induction), split crude extract after centrifugation (insoluble and soluble fraction), and eluted fractions from Ni-NTA column (2-43, marked in red). White asterisk indicates recombinant HMGB1. C) Chromatogram of pooled fractions 16-26 from A) on heparin Sepharose (100 Mol% NaCl equals 2 M NaCl concentration). The purification was followed by absorbance, collected fractions are indicated by red numbers. Fractions 29-31 were collected and used for ongoing experiments. D) The respective SDS-PAGE of selected fractions from the heparin column is depicted.

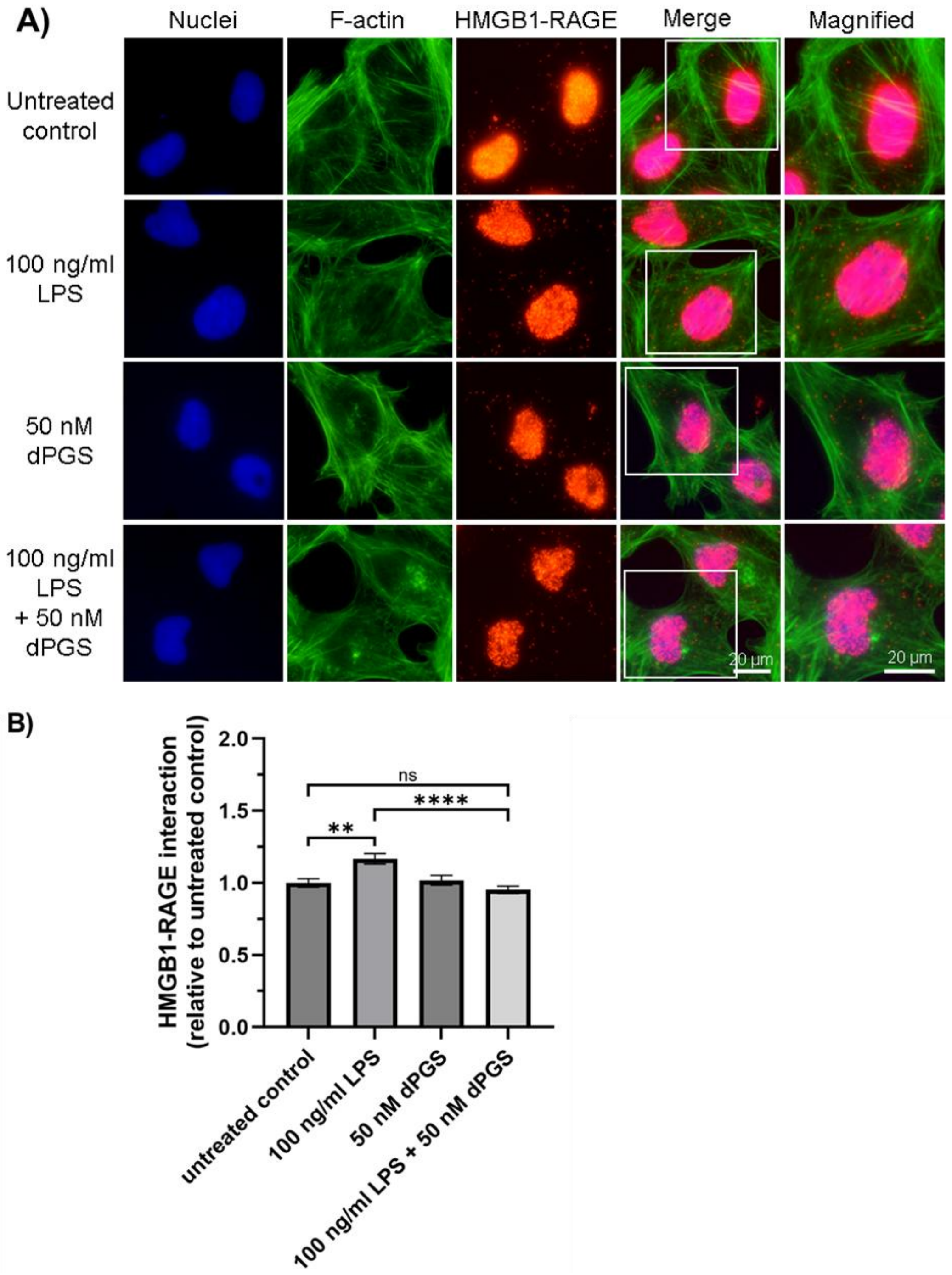

Figure S3. HMGB1/RAGE protein interaction in human microglia in response to dPGS and LPS. A) Fluorescence micrographs of HMGB1/RAGE interactions (red) in cells treated with dPGS (50 nM) with or without lipopolysaccharide (LPS, 100 ng/ml) for 24 hours, in serum-deprived medium. HMGB1/RAGE interactions were detected using a proximity ligation assay. Nuclei (blue) were labelled with Hoechst 33342, and F-actin was labelled with Alexa Fluor 488 Phalloidin. Scale bar = 20 µm. (B) Quantifications of HMGB1/RAGE interactions in cells treated as in A). Shown are the number of HMGB1/RAGE interactions per cell, as a fold change normalized the untreated control (set to 1). Shown are the mean ± SEM. 76 cells from three independent experiments were analyzed. Two-way ANOVA followed by Tukey´s multiple comparison test, ns = not significant, **p<0.01, ****p<0.0001.

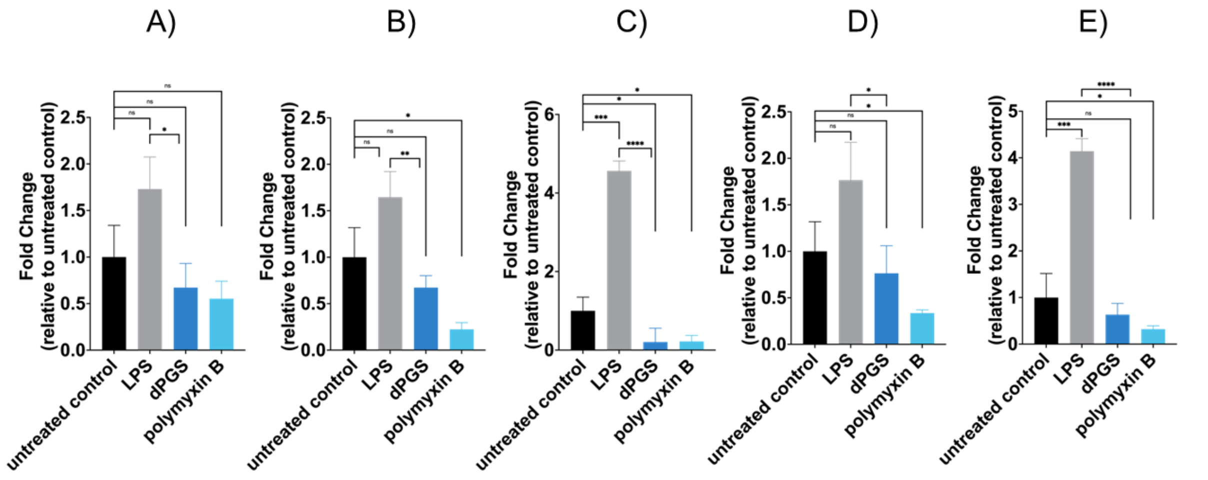

Figure S4. Real-time qPCR results. RT-qPCR results for A) MCP-1, B) IL-6, C) TNF-α, D) COX-2, and E) iNOS. The relative expression of the target genes was calculated using the $2^{-\Delta\Delta Ct}$ method using β-actin as the reference gene. Statistical analysis was done by using one-way ANOVA followed by Tukey´s multiple comparison test. Mean ± SEM. (****$p \leq 0.0001$, ***$p \leq 0.001$, **$p \leq 0.01$,*$p \leq 0.05$, ns = not significant).

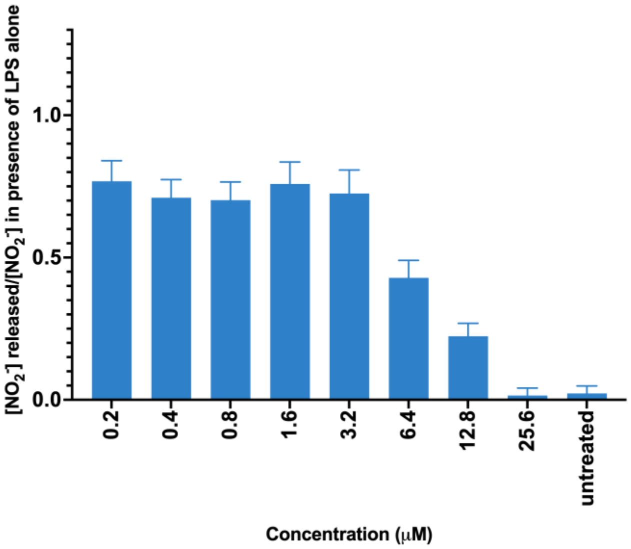

Figure S5. Concentration-dependent NO release from RAW 264.7 cells, as measured in the form of nitrite using Griess reagent. The concentrations are represented relative to the value measured for LPS (*E. coli*) alone. "Untreated" refers to cells not exposed to LPS, nor dPGS, while the other data points represent exposure to both LPS and the compound. Shown are the mean ± SEM.

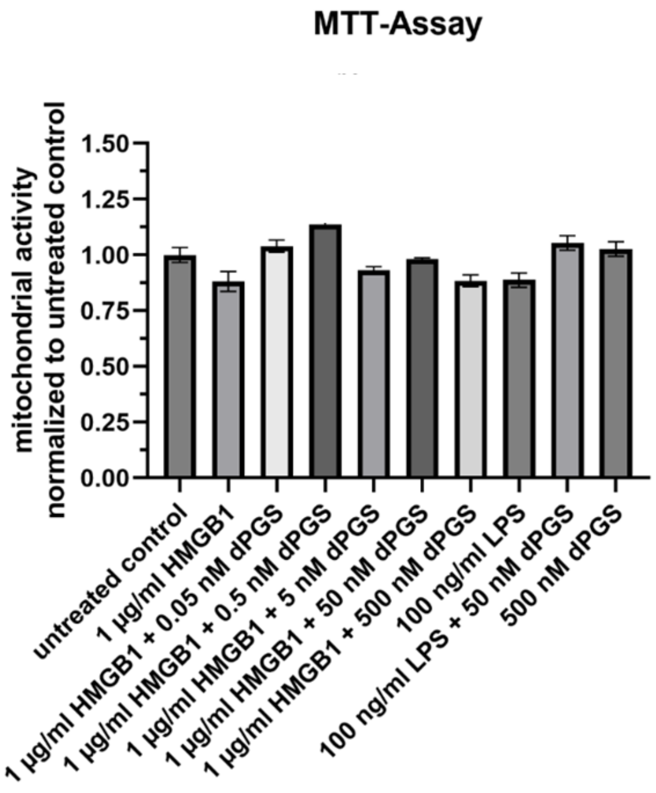

Figure S6. dPGS is a nontoxic compound. RAW 264.7 cells were seeded onto a 96-well plate with 10,000 cells/well in 1 x DMEM, 10 % FCS, 1 % Pen/Strep in 100 µL for 24 h. After washing once with 1 x DPBS, cells were stimulated with 100 µL of dPGS in concentrations of 500 nM, 50 nM, 5 nM, 0.5 nM, and 0.05 nM under serum-free conditions for 16 h. The supernatant was removed, and the cells were washed once more with 1 x DPBS before adding 100 µL of 0.5 mg/mL MTT in 1 x DMEM. Cells were incubated for 1 h, and formazan crystals were dissolved with DMSO for 15 min under moderate shaking at room temperature. The absorbance was measured at 595 nm, and the results are represented as fold change compared to the untreated control. The mean ± SEM is shown here. Experiments were conducted in triplicates and repeated three times.

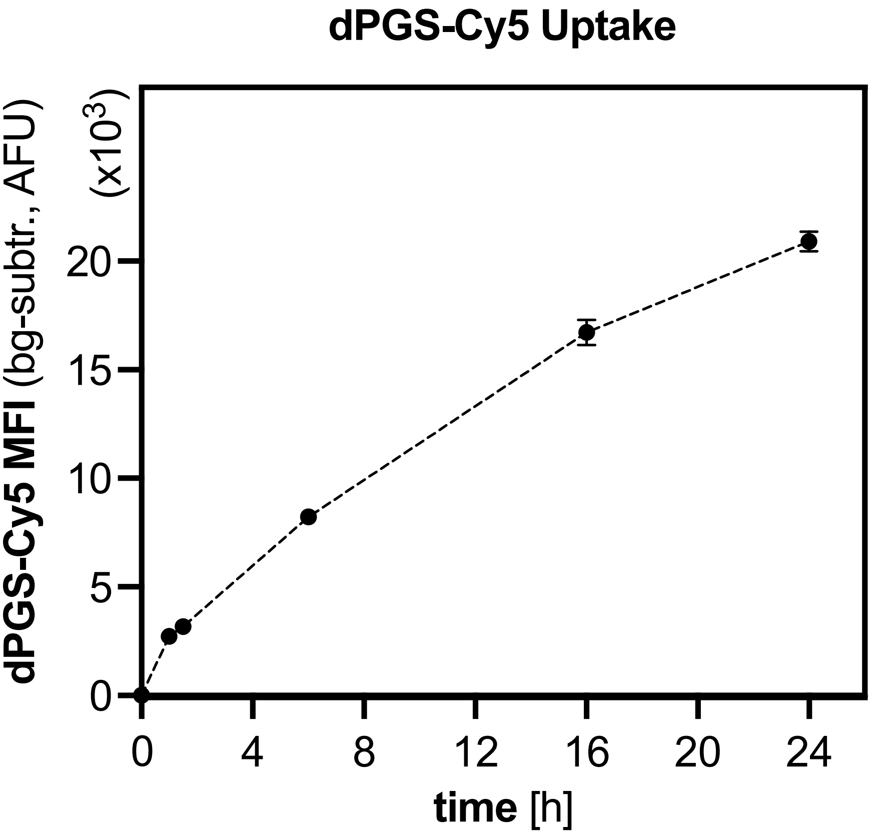

Figure S7. Time-dependent uptake of dPGS-Cy5 into RAW 264.7 macrophages. 1 x 10^5 cells/well were seeded in 24-well plates and incubated with 50 nM dPGS-Cy5 in serum-free medium for 1, 1.5, 6, 16, and 24 h. The cellular uptake of dPGS-Cy5 was quantified by flow cytometry and is given as background-subtracted MFI of Cy5. The data are shown as mean + SEM from three independent experiments, each performed with at least three technical replicates. bg-subtr. = background-subtracted, AFU = arbitrary fluorescence units.
